# Pediatric melanoma in Brazil: an analysis of mortality^؆^

**DOI:** 10.1016/j.abd.2025.501238

**Published:** 2025-11-01

**Authors:** Karina Munhoz de Paula Alve Coelho, José Guilherme Jasper Pickler, Camila Barbosa, Francis Rosseti Pedack, Bruna Louise Silva, Rafael Roesler, Paulo Henrique Condeixa de França, Hercilio Fronza Junior, José Cândido Caldeira Xavier-Junior

**Affiliations:** a Centro de Diagnósticos Anátomo-Patológicos, Joinville, SC, Brazil; b Instituto Nacional de Ciências e Tecnologia em Biologia do Câncer Infantil e Oncologia Pediátrica, Porto Alegre, RS, Brazil; c Postgraduate Program in Health and Environment, Universidade da Região de Joinville, Joinville, SC, Brazil; d Department of Teaching and Research, Hospital Municipal São José, Joinville, SC, Brazil; e Departament of Mechanical Engineering, Center for Technological Sciences, Universidade Estadual de Santa Catarina, Joinville, SC, Brazil; f Department of Pharmacology, Instituto de Ciências Básicas da Saúde, Universidade Federal do Rio Grande do Sul, Porto Alegre, RS, Brazil; g Cancer and Neurobiology Laboratory, Experimental Research Center, Hospital de Clínicas de Porto Alegre, Universidade Federal do Rio Grande do Sul, Porto Alegre, RS, Brazil; h Faculdade de Medicina, Centro Universitário Católico Unisalesiano, Araçatuba, SP, Brazil; i Instituto de Patologia de Araçatuba, Araçatuba, SP, Brazil

Dear Editor,

Cutaneous melanoma in children and adolescents is a rare condition, accounting for 1% to 3% of all pediatric malignancies and approximately 1% to 4% of all melanoma cases.^1^ The global incidence is estimated at 2 to 5 new cases per million people annually, with an average annual increase of 2% over the past few decades.^2^, ^3^, ^4^ However, recent data from the United States show a decreasing trend in new melanoma cases since 2006, particularly among adolescents.^3^ In the United States, 79% of cases in recent decades have been diagnosed in adolescents, while only 0.3% to 0.4% occur during the first decade of life.^5^

The risk factors, clinical presentation, and prognosis differ significantly among neonatal melanoma, childhood melanoma, and adult melanoma.^6^, ^7^, ^8^ Despite its rarity, pediatric melanoma is the most common skin cancer in children and a crucial diagnosis in pediatric oncology and dermatology, as melanocytic lesions are among the most frequent reasons for dermatological consultations in this age group.^7^, ^9^

Given its uncommon occurrence, the literature on pediatric melanoma remains limited, with most knowledge derived from single-institution studies or small regional cancer registries. Here, the authors describe mortality associated with melanoma in the pediatric population of Brazil.

The authors carried out a retrospective, descriptive exploratory analysis of melanoma mortality in the Brazilian pediatric population, using nonidentifiable national registry data from the Mortality Information System (SIM), provided by the Department of Informatics of the Brazilian Unified Health System (DATASUS). Consolidated data from 2008 to 2022, including preliminary data from 2023, were analyzed. The processing and coding of mortality data followed the official documentation provided by DATASUS, and gathered from the official File Transfer Protocol (FTP) address (ftp.datasus.gov.br), with the needed data decompression and conversion from dbc (compressed 'XBASE' database) into comma-separated values. The inclusion criteria established were underlying causes classified under group C43 of the International Classification of Diseases (ICD-10) and age at death between 0 and 19 years and 364 days. The variables extracted from SIM reflect the Death Certificate (DO), and for this research, they include year and date of death, date of birth, age at death, gender, race, federal unit, underlying cause, and items A, B, C, and D from the DO. Data collection was carried out on 09/24/2024, based on the latest publicly available version. Demographic information and population indices were obtained through the Application Programming Interface (API) of the Brazilian Institute of Geography and Statistics (IBGE), integrating census data and official population estimates. The average mortality rate was calculated for the total pediatric population (0 to 19 years) and for the age groups of 0 to 9 years and 10 to 19 years, using data from IBGE between 2008 and 2023. To calculate the annual mortality rate, the number of deaths was divided by the total population, and the result was multiplied by 100,000. To obtain the average mortality rate, the annual mortality rates were accumulated, and the total was divided by the 16 years of the period. Power BI was used for the creation of graphs.

A total of 113 cases of melanoma-related deaths in pediatric patients in Brazil were identified from 2008 to 2023, corresponding to 0.01% of all deaths in the pediatric population. Fig. 1 shows the distribution of deaths per year. The distribution of deaths by age per year shows a predominance in the adolescent population (84, which corresponds to 74%), compared to children (29, or 26%) (Fig. 2). The average mortality rate was 0.011367 per 100,000 inhabitants for the total pediatric population (0 to 19 years), with 0.00613 for the 0 to 9 years age group and 0.014785 for the 10 to 19 years age group.

**Fig. 1:**
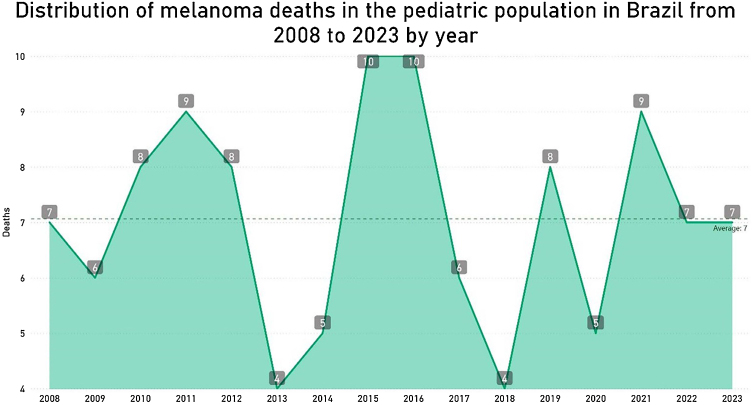
Distribution of melanoma deaths per year in the pediatric population in Brazil from 2008 to 2023. The number of deaths peaked in 2015 and 2016, with 10 deaths per year. Other years with relatively high numbers were 2011 and 2021, with 9 deaths each. Most years showed relatively low and consistent numbers of deaths, ranging from 4 to 8.

**Fig. 2:**
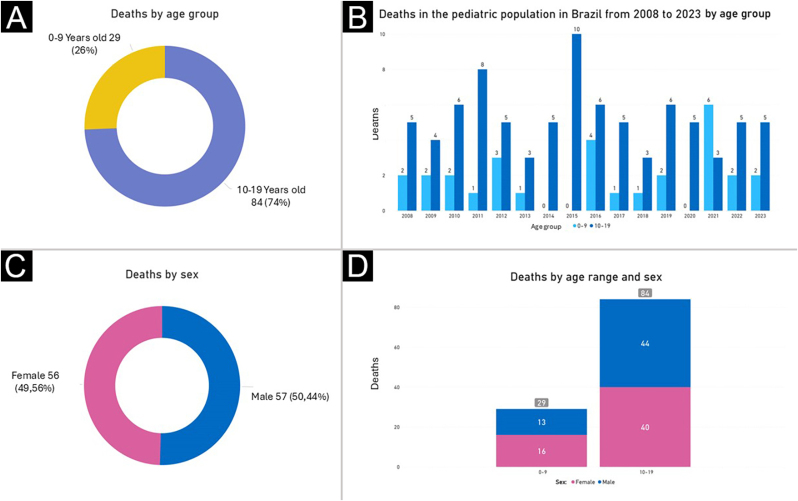
Distribution of melanoma deaths in the pediatric population in Brazil from 2008 to 2023, by age and sex. (A) Overall distribution of deaths by age group, with the 0‒9 years group representing 26% of total deaths (29 deaths), while the 10‒19 years group accounts for 74% (84 deaths). (B) Distribution of deaths by age group over the years. The 10‒19 years group, represented in dark blue, frequently shows higher numbers of deaths than the 0‒9 years group (light blue). However, distinct peaks were observed in specific years. In 2015, the 10‒19 years group recorded the highest number of deaths, with 10 deaths, while in 2021, the 0‒9 years group had the highest number of deaths, with 6 deaths. Although the 10‒19 years group has a generally higher prevalence of deaths, the annual variations in the 0-9 years group suggest a non-linear distribution of deaths over the years. (C) Overall distribution of deaths by sex, with 49.56% of deaths occurring in females (56 deaths) and 50.44% in males (57 deaths). (D) Number of deaths by age group and sex, with 13 (45%) deaths in males and 16 (55%) in females in the 0‒9 years age group (totaling 29 deaths), and 44 (52%) deaths in males and 40 (48%) in females in the 10‒19 years age group (totaling 84 deaths). These data indicate a generally balanced distribution of deaths between the sexes, with a slight predominance of females in the 0‒9 years age group and a nearly equal distribution in the 10‒19 years age group.

There was a small predominance in the male population (50.44%). However, when analyzed by age group, there were more deaths among females in children and among males in adolescents (Fig. 2D). These findings indicate the need for equitable attention to both sexes in prevention and early diagnosis strategies. The authors also analyzed the distribution of death cases for each region and state in Brazil. Results showed a predominance of deaths from pediatric melanomas in the southeast (32.74%) and southern (31.86%) regions and in the states of São Paulo (n = 20) and Rio Grande do Sul (n = 19) (Fig. 3 and Fig. 4). There was also a higher number of records in the white-skinned population, followed by the mixed-race (brown) population (Fig. 4). These findings may be related to population density, better quality of notification data, easier access to health services and the higher proportion of light-skinned individuals, who are generally at greater risk for melanoma. The Brazilian phenotypic distribution exhibits considerable heterogeneity across latitudes. In the states of Santa Catarina and Rio Grande do Sul, 89% of the urban population has fair skin.^10^

**Fig. 3:**
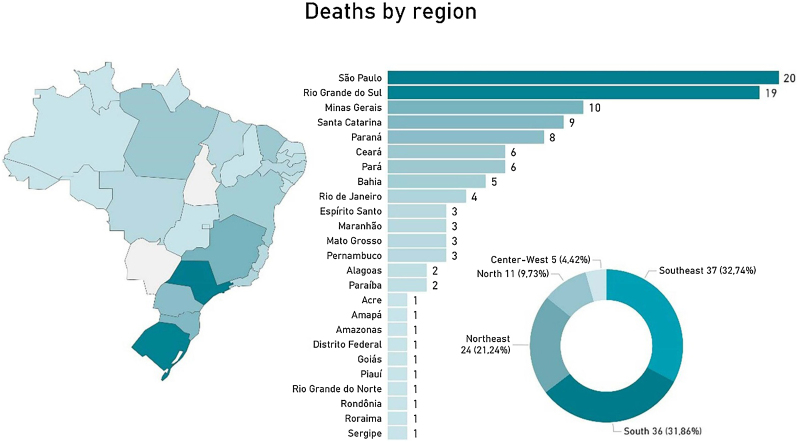
Distribution of melanoma deaths in the pediatric population in Brazil from 2008 to 2023, by state and region. The map on the left shows the geographic distribution of melanoma deaths, with states reporting the highest numbers of deaths represented in darker green. The bar graph in the center presents the total number of deaths by state and the Federal District, with São Paulo (20 cases) and Rio Grande do Sul (19 cases) at the top of the graph. The pie chart in the lower left shows the distribution of deaths by region, with the southern (36 cases, 31.86%) and southeastern (37 cases, 32.74%) regions reporting the highest numbers of deaths, followed by the northeast (24 cases, 21.24%), northern (11 cases, 9.73%), and central western regions (5 cases, 4.42%). The darker green colors in all three graphs indicate the areas with the highest number of deaths.

**Fig. 4:**
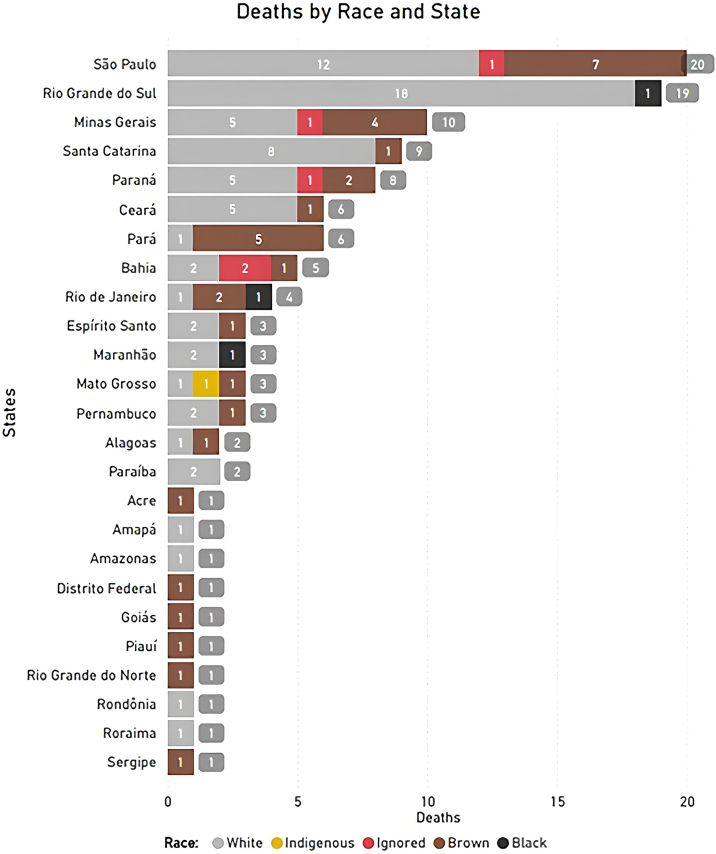
Distribution of melanoma deaths in the pediatric population in Brazil from 2008 to 2023, by race and state. The bar graph presents the number of deaths by state and race, with the following color coding: gray for white, yellow for indigenous, red for ignored (unknown), brown for brown (mixed-race), and black for black. The data indicate that white children represent the majority of deaths, with São Paulo, Rio Grande do Sul, and Santa Catarina showing the highest numbers in this group. Black and indigenous children show fewer deaths, with only a few states reporting cases. Brown (mixed-race) children also account for a significant portion of deaths, with a more even distribution across various states.

The main limitations of the present study are related to the retrospective nature of the database and the analysis conducted on raw data, which were not adjusted for the population. The national data did not provide important histopathological features such as histological subtype, presence of ulceration, and staging. In addition, the results were based on secondary data, and the actual number of deaths by melanoma in this age group might be higher. Furthermore, data considering the distinct clinical forms of pediatric melanoma were not available.

In summary, this study reports deaths from melanoma in the pediatric population in Brazil, highlighting the need for specific strategies for prevention, early diagnosis, and treatment. The clinical management of pediatric melanoma is challenging and lacks specific guidelines, making it crucial to ensure access to clinical trials and new therapies that can alter the natural history of advanced melanoma. Future studies should preferably include data on predisposing conditions, such as xeroderma pigmentosum or congenital melanocytic nevi, to enhance the understanding of the underlying etiology and improve risk stratification strategies in this vulnerable population.

## ORCID ID

José Guilherme Jasper Pickler: 0009-0003-9291-8109

Camila Barbosa: 0000-0003-3112-5781

Francis Rosseti Pedack: 0009-0007-9382-5042

Bruna Louise Silva: 0000-0001-9756-4846

Rafael Roesler: 0000-0001-6016-2261

Paulo Henrique Condeixa de França: 0000-0002-175 0-9 132

Hercilio Fronza Junior: 0000-0002-0353-0926

José Cândido Caldeira Xavier Junior: 0000-0003-0503-419X

## Financial support

This work was supported by the National Council for Scientific and Technological Development (CNPq, MCTI, Brazil), grant number 406484/2022-8 (INCT BioOncoPed) to R.R., and by the Centro de Diagnósticos Anátomo-Patológicos (CEDAP, Brazil). R.R. is also supported by CNPq grant numbers 305647/2019-9 and 405608/2021-7, the Children’s Cancer Institute (Instituto do Câncer Infantil, ICI), and institutional research funds from Hospital de Clínicas de Porto Alegre (HCPA). P.H.C.F is also supported by CNPq grant number 307616/2023-1.

## Authors’ contributions

Karina Munhoz de Paula Alves Coelho: Data collection, or analysis and interpretation of data; drafting and writing of the manuscript; final approval of the manuscript; study supervision.

José Guilherme Pickler: Data collection, or analysis and interpretation of data; final approval of the manuscript.

Camila Barbosa: Critical review of the literature; drafting and writing of the manuscript; final approval of the manuscript.

Francis Rosseti Pedack: Data collection, or analysis and interpretation of data; final approval of the manuscript.

Bruna Louise Silva: Final approval of the manuscript.

Rafael Roesler: Drafting and writing of the manuscript; final approval of the manuscript.

Paulo Henrique Condeixa de França: Drafting and writing of the manuscript; final approval of the manuscript.

Hercilio Fronza Junior: Data acquisition, analysis, and interpretation; final approval of the manuscript.

José Cândido Caldeira Xavier Junior: Study conception and design; study supervision; final approval of the manuscript.

## Research data availability

The entire dataset supporting the results of this study was published in this article.

## Conflicts of interest

None declared.
